# Characterization of Breast Cancer Intra-Tumor Heterogeneity Using Artificial Intelligence

**DOI:** 10.3390/cancers16223849

**Published:** 2024-11-16

**Authors:** Ayat G. Lashen, Noorul Wahab, Michael Toss, Islam Miligy, Suzan Ghanaam, Shorouk Makhlouf, Nehal Atallah, Asmaa Ibrahim, Mostafa Jahanifar, Wenqi Lu, Simon Graham, Mohsin Bilal, Abhir Bhalerao, Nigel P. Mongan, Fayyaz Minhas, Shan E Ahmed Raza, Elena Provenzano, David Snead, Nasir Rajpoot, Emad A. Rakha

**Affiliations:** 1 Breast Cancer Research Unit, University of Nottingham, Nottingham NG7 2RD, UK; ayatlashen133@gmail.com (A.G.L.); michaelsalah85@yahoo.com (M.T.); islam.abdelaziz@nhs.net (I.M.); mzxsg2@exmail.nottingham.ac.uk (S.G.); mzxsm9@exmail.nottingham.ac.uk (S.M.); mzxna4@exmail.nottingham.ac.uk (N.A.); mzxai2@exmail.nottingham.ac.uk (A.I.); 2 Department of Pathology, Faculty of Medicine, Menoufia University, Shibin El-Kom 6131567, Egypt; 3 Department of Computer Science, University of Warwick, Coventry CV4 7AL, UK; noorul.wahab@warwick.ac.uk (N.W.); mostafa.jahanifar@warwick.ac.uk (M.J.); wenqi.lu.1@warwick.ac.uk (W.L.); simon.graham@warwick.ac.uk (S.G.); mohsin.bilal@warwick.ac.uk (M.B.); abhir.bhalerao@warwick.ac.uk (A.B.); fayyaz.minhas@warwick.ac.uk (F.M.); shan.raza@warwick.ac.uk (S.EA.R.); david.snead@pathlake.org (D.S.); n.m.rajpoot@warwick.ac.uk (N.R.); 4 School of Veterinary Medicine and Sciences, University of Nottingham, Nottingham LE12 5RD, UK; svznpm@exmail.nottingham.ac.uk; 5 Department of Pharmacology, Weill Cornell Medicine, New York, NY 10065, USA; 6 Department of Pathology, Cambridge Biomedical Research Centre, Cambridge University Hospitals, Cambridge CB2 0QQ, UK; elenaprovenzano@addenbrookes.nhs.uk; 7 Department of pathology, University Hospital Coventry and Warwickshire, Coventry CV2 2DX, UK; 8 Pathology Department, Hamad General Hospital, Hamad Medical Corporation, Doha P.O. Box 3050, Qatar

**Keywords:** breast cancer, intra-tumor heterogeneity, artificial intelligence

## Abstract

Intra-tumor heterogeneity (ITH) is a fundamental characteristic of breast cancer (BC), influencing tumor progression, prognosis, and therapeutic responses. However, the complexity of ITH in BC makes its accurate characterization challenging. This study leverages deep learning (DL) techniques to comprehensively evaluate ITH in early-stage luminal BC and provide a nuanced understanding of its impact on tumor behavior and patient outcomes. A large cohort (*n* = 2561) of early-stage luminal BC was evaluated using whole slide images (WSIs) of hematoxylin and eosin-stained slides of excision specimens. Morphological features of both the tumor and stromal components were meticulously annotated by a panel of pathologists in a subset of cases. A DL model was applied to develop an algorithm to assess the degree of heterogeneity of various morphological features per individual case utilizing defined patches. The results of extracted features were used to generate an overall heterogeneity score that was correlated with the clinicopathological features and outcome. Overall, 162 features were quantified and a significant positive correlation between these features was identified. Specifically, there was a significant association between a high degree of intra-tumor heterogeneity and larger tumor size, poorly differentiated tumors, highly proliferative tumors, tumors of no special type (NST), and those with low estrogen receptor (ER) expression. When all features are considered in combination, a high overall heterogeneity score was significantly associated with parameters characteristic of aggressive tumor behavior, and it was an independent predictor of poor patient outcome. In conclusion, DL models can be used to accurately decipher the complexity of ITH and provide extra information for outcome prediction.

## Simple Summary

The emerging era of digital pathology and Artificial Intelligence provides a better tool for the assessment of multiple morphological features extracted from scanned histological images. In this study, we aimed to develop an AI-based algorithm to measure and quantify features that represent intra-tumor heterogeneity in breast cancer and present them as an index. In our study, 162 features were extracted and quantified from whole slide images. These features showed a significant association with patient outcomes. When an overall heterogeneity score was used, it stratified luminal breast cancer patients into low- and high-risk groups, which can help in personalized therapy.

## 1. Introduction

Breast cancer (BC) is a heterogeneous disease with variable tumor characteristics and patient outcomes [1]. In addition to the inter-tumor heterogeneity that distinguishes patients with BC, each tumor also exhibits genetic, immunophenotypic, and morphologic variability in distinct tumor foci, termed intra-tumoural heterogeneity (ITH) [2]. ITH describes the presence of various cell subpopulations, including tumor and stromal cells that are distributed throughout the tumor areas. These subpopulations differ in their cellular and biologic properties within a specific primary tumor location (spatial heterogeneity) and between the primary tumor and its metastases (temporal heterogeneity) [1,3].

BC is well known to have significant spatial ITH, which can be represented in two categories: histologic and functional ITH [4,5]. The term “histologic ITH” refers to the morphological tumor features that can be assessed using histopathologic examination such as histologic tumor type, tumor differentiation, architecture, cellularity, stromal response, and immunologic response [5]. Functional ITH refers to differences in the genetic makeup of the malignant cells within the same tumor and determines their behavior [5] and these can be assessed using various molecular techniques such as single-cell RNA sequencing (scRNA-seq). However, assessing ITH in BC through genetic or epigenetic assays can be quite challenging in routine clinical practice [1,6]. Traditional methods often involve complex genetic analysis of micro-dissected multi-regions from the same tumor, which is difficult to replicate consistently [7]. These methods can also produce a lot of noise, making it hard to obtain clear results. Contrasting this, the morphologic features of the tumor represent the end product of the functional activity of thousands of genes working together to determine tumor phenotype [8].

Morphologic heterogeneity is documented in several studies but remains challenging to assess in clinical practice. Theoretically, assessment of histologic ITH could be performed visually from the whole slide in a way similar to the assessment of ITH in the expression of Ki67 [9] estrogen receptor (ER) [10] PDL1, and tumor-infiltrating lymphocytes (TILs) [11,12,13,14,15,16]. However, such visual assessment is still limited by the capacity of pathologists to assess the complex cellular and spatial distribution of often subtle features or to integrate multiple features that represent ITH in a meaningful score that can be applied in clinical practice [17]. In addition, the molecular subtype of BC can potentially impact the assessment of ITH [18].

The emerging era of digital pathology and rapid development of deep learning (DL) methodologies have enabled fast and automatic extraction of clinically relevant information from whole-slide images (WSI) [19]. In addition, Artificial Intelligence (AI)-based tools for high throughput analysis of complex morphologic features retrieved from WSI provide avenues for an objective and standardized approach to the assessment of tumor-related morphologic features beyond the capacity of the human eye such as ITH. Furthermore, AI-based algorithms enable the quantification of complex features of tumor phenotype in the spatial context of the tissue, which is imperative for ITH assessment [8]. AI-based methods have shown promise in quantifying intra-tumor heterogeneity (ITH) for various downstream tasks. For instance, texture features from predicted spatial gene heatmaps have been found to provide independent prognostic value for BC [20,21]. Additionally, DL-based cell segmentation and classification have been used to model spatial immune infiltration, classifying tumor regions into immune hot and cold subtypes, which correlate with relapse risk in lung cancer [22]. Another ITH study based on hexagonal grid analytics enabled the quantification of proliferation ITH in BC [23]. However, the challenge of lacking ground truth for ITH assessment remains significant. Many methods that aim to detect and quantify subvisual features cannot rely on ground truth data obtained through visual assessment [24].

In this study, we investigated ITH, using AI and digital pathology technology for interpretable features, in a large cohort of luminal (ER and progesterone receptor (PR) positive and human epidermal growth factor 2 (HER2) negative) BC to eliminate the impact of molecular subtype on the morphologic features of the tumors. To address the challenge of interpreting features from DL-based models, the proposed AI pipeline integrates well-established domain-specific features, such as co-occurrence and contrast, to enhance interpretability. This study provides evidence that ITH can be deciphered using AI to further refine BC prognostic stratification that can be applied in a routine clinical setting.

## 2. Material and Methods

### 2.1. Study Cohort and Image Acquisition

This study was carried out using a large cohort (*n* = 2561) of ER-positive and HER2-negative BC patients presented at Nottingham University Hospitals, Nottingham, UK. The clinicopathologic data for this cohort included the patient’s age, tumor size, histological grade, histological tumor type, lymph node (LN) status, lymphovascular invasion (LVI), and Nottingham prognostic index (NPI) (Supplementary Table S1). In this study, we chose only a cohort of luminal BC, which is considered a challenging group of BCs with indeterminate recurrence risk. We have selected this uniform cohort (ER+/HER2−), which is less likely to be heterogenous and to eliminate the impact of molecular classes because HER2+ and triple-negative BC have different morphology.

The median follow-up time was 138 months with available outcome data, including BC-specific survival (BCSS), defined as the time (in months) from the date of the primary surgery to the time of death from BC, and distant metastasis-free survival (DMFS), defined as the time (in months) from the primary surgery until the first event of distant metastasis.

Out of this cohort, 1933 patients showed no LN invasion (LN^0^), while 628 patients showed LN stage 2 (1–3 involved nodes). A total of 93% of patients in this cohort received adjuvant endocrine therapy and only 7% received both endocrine therapy and chemotherapy. Biomarker expression data including PR and Ki67 were also available using full face sections as previously described [25,26,27]. Hematoxylin and eosin (H&E)-stained slides were scanned into high-definition digital images by high-resolution scanning (0.19 mm/pixel) with a high throughput scanner (Panoramic 250 Flash III; 3DHistech, Budapest, Hungary).

### 2.2. Algorithm Training

**Figure 1:**
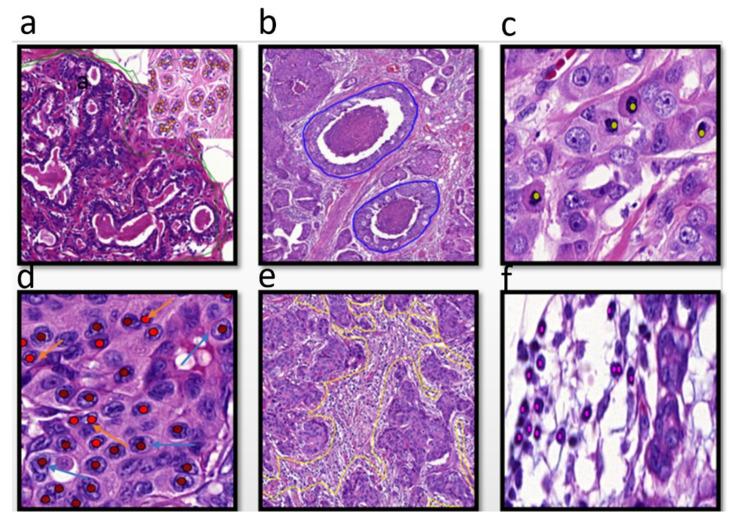
The different annotated morphological features at 40×, (**a**) normal glands (annotated with green lines), (**b**) ductal carcinoma in situ, annotated with blue lines, (**c**) mitotic figures, annotated with light green dots (**d**) different pleomorphic cells, annotated with red dots, (**e**) tumor-associated stroma, annotated with yellow lines, (**f**), tumor-infiltrating lymphocytes (TILs) annotated with pink dots.

In order to identify the morphologic features of tumors for assessing ITH, a group of qualified pathologists thoroughly annotated different morphological features of BC using a large, sizeable subset of BC cases to train the detection algorithm. The annotated tumor features included tumor grade components (tubule formation, degree of polymorphism in tumor cell nuclei, and mitotic figures), ductal carcinoma in situ (DCIS) regions, different components of the tumor microenvironment (TME), such as tumor-associated stroma, which lay immediately adjacent to the cancer cells, fibrofatty stroma, and both intra-tumor and stromal (TILs) [28,29] (Figure 1).

### 2.3. Deep Learning-Based Feature Computing

**Figure 2:**
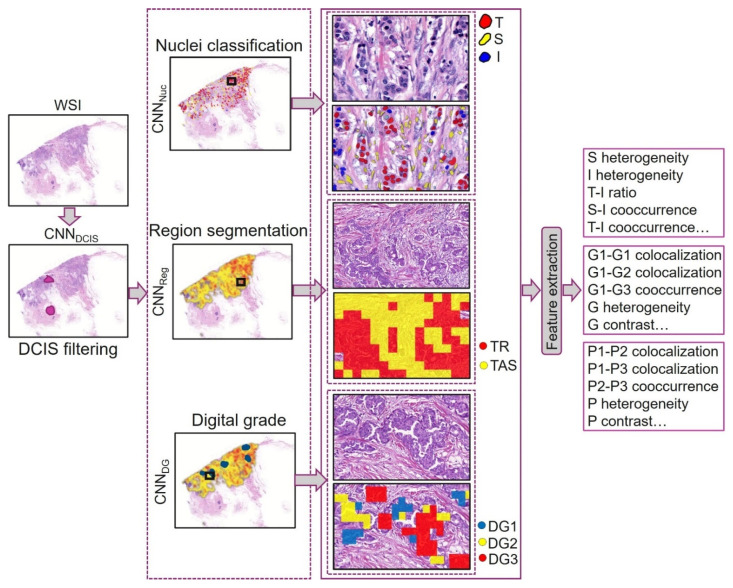
Deep learning (DL)-based morphological feature extraction: A convolutional neural network (CNN), CNN_DCIS_, first filters out ductal carcinoma in situ (DCIS). Next, CNN_Nuc_ segments and classifies nuclei, followed by CNN_Reg_, which segments regions. CNN_DG_ assigns digital grade to tissue. The outputs from nuclear classification, regional segmentations, and digital grading are utilized to compute a range of morphological features. S: stroma, I: immune, T: tumor, G: grade, P: pleomorphism, TR: tumor region, TAS: tumor-associated stroma.

A deep learning (DL) pipeline was developed to extract morphological features of tumors and stroma (Figure 2). Thresholding and morphological operations were applied to create a tissue mask, ensuring that only the relevant tissue areas were processed in subsequent steps. This step eliminates background regions, artifacts, or non-tissue areas, allowing the model to focus solely on the tissue for further analysis, including DCIS filtering and feature extraction. A convolutional neural network (CNN)-based model (CNN_DCIS_) was employed to exclude DCIS regions from the tissue followed by CNN_Reg_ to segment tumors, stroma, and other tissue. CNN_Nuc_ was used to segment and classify different nuclear types, with tumor and stromal nuclei being used to derive nuclear-related features. Similarly, CNN_DG_ predicted digital grade and pleomorphism for patches of a WSI.

#### 2.3.1. DCIS and Other Region Segmentation

To exclude DCIS regions from downstream analysis, an efficient U-Net-based semantic segmentation model (CNN_DCIS_) [20], trained on pathologist-annotated tumors and DCIS regions, was applied to the tissue. The areas identified as DCIS were removed from all subsequent processing steps.

The U-Net model [5] (referred to as CNN_Reg_) was modified by adding two additional encoding and decoding blocks for the semantic segmentation of tumor, stromal, and other non-ROI regions, and trained using pathologist-annotated regions. The training parameters were as follows: patch size of 512 × 512 pixels with 96-pixel context on all sides; learning rate starting at 0.01 for the first five epochs, reduced to 0.001 for epochs 6–10, and further to 0.0001 for epochs 11–30; momentum set to 0.9; batch size of 8; and cross-entropy loss function. Additional settings included normalizing the input to {0, 1} and applying various augmentations (random rotation, random brightness, and median blur) with a probability of 0.5.

#### 2.3.2. Nuclei Segmentation and Classification

**Figure 3:**
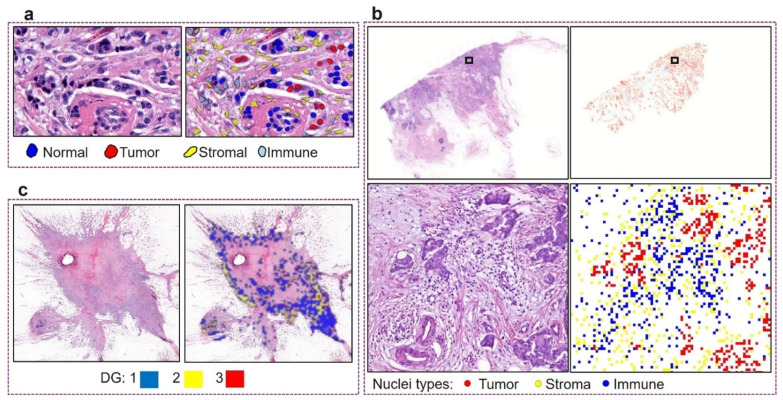
Various features generated from the deep learning models. (**a**) Qualitative results of nuclear segmentation and classification from CNN_Nuc_; (**b**) generating a five-times down-sampled WSI cell maps for texture feature calculation; (**c**) qualitative results of digital grade prediction from CNN_DG_.

A custom-built nuclear segmentation and classification model, HoVer-Net, was used to identify and categorize different types of nuclei [21]. The model, initially pretrained on the breast cancer subset of the PanNuke dataset, was further fine-tuned for the target dataset, resulting in CNN_Nuc_. The fine-tuning process used a patch size of 256 × 256 pixels, with a learning rate of 0.0001 for the first two epochs, followed by 0.00001 for subsequent epochs, and a batch size of 8. Figure 3a shows some qualitative results of nuclear segmentation and classification. During inference on a WSI, the nuclear mask was created by assigning each detected nucleus to its corresponding type or class at the nucleus centroid in a five-times down-sampled WSI (Figure 3b).

#### 2.3.3. Tumor Differentiation (Digital Grade Prediction)

To build a model for predicting tumor grade and pleomorphism from an input patch, we enhanced the Inception V3 network [30] by adding two linear layers with ReLU activation, along with a fully connected layer referred to as CNN_DG_. Pretrained ImageNet weights [31] were retained for the unchanged layers. For training, one ROI per WSI from the discovery set of each split was selected, and model selection was based on performance on the validation set. ROIs measuring 5600 × 5600 pixels at 40× magnification were selected based on tumor cell density. CNN_DG_ was trained using a batch size of 8, a learning rate of 0.001, a momentum of 0.9, and a cross-entropy loss function. Inputs were normalized using ImageNet statistics, and various augmentations from the torchvision library were applied, including random cropping, random horizontal flips (*p* = 0.5), and color jittering (brightness, contrast, saturation, and hue adjustments set to 0.3). The trained model was later used to predict digital grade and pleomorphism for the entire WSI on a patch-by-patch basis (Figure 3).

### 2.4. Defining the Morphological Features to Assess ITH

**Figure 4:**
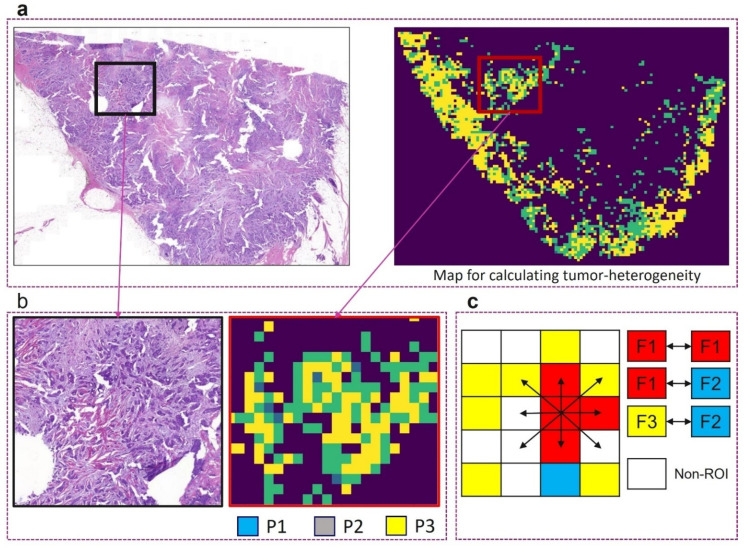
Sample heterogeneity feature calculation. (**a**) A WSI along with digital pleomorphism (P1-3) prediction from CNN_DG_; (**b**) enlarged ROIs from panel (**a**); (**c**) example of co-occurrence matrix for three features (F1-3) showing the eight directions used for features calculation.

From the available annotated data and the DL pipeline, features related to tumor and stroma were defined and calculated for each patient to assess ITH. These features were extracted from several patches (each is 62 µm^2^ size) from each of the WSI (Figure 3 and Figure 4). By computing a number of variables and producing a co-occurrence matrix (CM), Haralick texture indicators can quantify morphological features that represent ITH in the form of uniformity measures and spatial entropy, which integrates proximity between features as a key spatial component into ITH measurement [32]. Features were calculated using a standard Python library Scikit-image [33]. These features are mainly related to tumor differentiation, TME, which including stroma, and TIL heterogeneity. For each feature, several measures were calculated and recorded; colocalization represents the percentage of each feature in an area. A colocalization value of 0 means the features do not colocalize, whereas a value of 1 means the same percentage of the features found together [33]. The total number of co-occurrences (representing the frequency of each component of these features in close proximity to other components) was divided by 2 and these values were then normalized (i.e., each value is divided by the total sum of the values), and the resulting values were termed as CM. CM was used to calculate different features including homogeneity and heterogeneity features such as contrast, dissimilarity, and correlation as previously described [34,35]. The sum of squared elements in the matrix, also known as uniformity, which ranged between 0 and 1, was also calculated where a contrast of 0 means a constant image. Energy is quite similar to contrast but with a linear-dependent off-diagonal of the CM [36]. While homogeneity measures the closeness of the element’s distribution in the CM diagonally, heterogeneity was calculated as (1—homogeneity) [37,38]. Tumor differentiation features were mainly related to tumor architecture, tubule formation, mitosis, nuclear, and cytologic features. The TME includes stromal features, (which were related to only the tumor associated stroma within the context of tumor cells). It also includes stromal and tumor TILs, their spatial distributions, and the co-occurrence between these features. Co-occurrence scores representing the frequency of each component of these features in close proximity to other components were also calculated. All measures of tumor differentiation features were normalized (0–100) and combined into one score. The same was performed for measures of stromal and TILs features. Supplementary Table S2 shows an overview of texture features employed in current study.

To develop an overall heterogeneity score in luminal BC, all extracted features related to tumor differentiation, stroma, and TILs were tested against BC outcomes in univariate analysis. Overall heterogeneity (ITH) score was calculated using multiple linear regression as follows [39]. Out of the 162 extracted features, 72 exhibited a significant association with patients’ outcomes. Then, a multivariate Cox regression-based analysis for each of the 72 significant variables was carried out. The overall heterogeneity score was calculated from the beta (b) value generated from each of the significant features in the multivariate analysis and multiplied by the feature values. Overall heterogeneity score = a + b1 × 1 + b2 × 2 + b3 × 3, etc. The overall heterogeneity score and the individual heterogeneity features were tested against the available clinicopathological data, outcome, and adjuvant therapy.

### 2.5. Statistical Analysis

Statistical Package for the Social Sciences software v.26.0 (SPSS, Chicago, IL, USA) was used for statistical analysis. The Chi-square test was used for the analysis of categorical data while the Pearson test was used to assess the correlation between continuous variables. Mean heterogeneity scores across tumor grades and other clinical parameters were compared by ANOVA test. To assess the best cut-off point to dichotomize the scores of ITH morphological features into low and high degree of heterogeneity, X-tile bioinformatics software version 3.6.1 (School of Medicine, Yale University, New Haven, CT, USA) [40] was used. Patients having values greater than the cut-off were deemed as the heterogeneous group, and, otherwise, they were considered as in the low heterogenous category.

For statistical analysis, Ki67 expression levels were categorized into low and high using a 10% cut-off [25,41]. For studying the association of heterogeneity with the levels of ER expression in tumors, ER expression levels were classified into 3 groups: lower ER < 10%, ER intermediate (11–99%), and highest expression (100%) [42]. PR expression levels 10% cut-off was used to categorize PR as low and high groups as previously defined [26,41]. X-tile was also used to determine the best cut-off, which was 4.9 to dichotomize the ITH score into low and high groups. Then, it was correlated with the available clinicopathological and outcome data. Considering the individual features of ITH, measures of each feature were combined into one score and this score was categorized into 2 groups based on X-tile for statistical analysis.

Outcome analysis was assessed using Kaplan–Meier curves and the log-rank test. A Cox regression model was used for multivariate analysis. The proportionality assumption of the Cox model for the ITH score was satisfied using the computed time-dependent covariate test.

## 3. Results

Overall, 162 features related to tumor morphology and TME were identified, extracted, and quantified from WSI patches in large early-stage luminal BC to assess ITH in BC using the DL model. These features were assessed individually, and then all features were compiled into one score. A significant positive correlation was found between morphological features of tumors (*p* < 0.0001). There was not only a positive correlation between tumor features but also between tumor and stromal features (*p* < 0.0001).

### 3.1. Performance of the DL Models

Table 1 lists the details of the validation of the DL models in terms of different metrics. Similarly, Table 2 lists the cell classification results of different models as compared to CNN_Nuc_. It can be observed that fine-tuning the pretrained model on the target data helped improve the results.

### 3.2. Association of ITH with Clinicopathologic Parameters

Considering the individual morphologic tumor features of ITH, the highest degree of intra-tumor differentiation heterogeneity was seen in histologic grade 3 (80%), and to a lesser degree in large-size tumors (54%) and in young premenopausal patients. Lower levels of ER expression and higher levels of Ki67 index were associated with an increased degree of intra-tumor differentiation heterogeneity (both *p* < 0.0001). Special tumor types showed the lowest levels of intra-tumor differentiation heterogeneity compared to no special type (NST) tumors (10% versus 45%, respectively). A total of 13% of invasive lobular carcinoma showed high levels of intra-tumor differentiation heterogeneity. The high degree of intra-tumor differentiation heterogeneity was associated with the development of LVI by approximately 2-fold (*p* < 0.0001) (Table 3).

The high degree of stromal heterogeneity was significantly associated with younger age, premenopausal status, larger tumor size, grade 3 tumors, NST tumors, LVI, and high Ki67 expression (all *p* < 0.0001). Similarly, the high degree of TIL heterogeneity showed a strong association with clinical parameters characteristic of aggressive tumor behavior (Table 4).

### 3.3. ITH Score

The mean ± standard deviation (SD) of the ITH was (10.4 ± 25.1), the median was 2.5 and ranged from (−14.25 to 195.87). A low ITH score (≤4.9) was found in 59% (1039/1774) of patients, while a high ITH score was observed in 41% (735/1774) of patients.

The mean ± SD of ITH score in grade 3 tumors was 15.4 ± 15.7 compared to 2.9 ± 17.9 in grade 1 tumors and this difference was statistically significant (*p* < 0.0001). A significant difference in the mean ITH score was observed between grade 2 and grade 3 (*p* = 0.03). This indicates a strong positive correlation between tumor grade and ITH scores with an incremental increase in ITH scores with higher grades. With regards to tumor size, there was a significant difference in the mean ITH score between larger and smaller (<2 cm) tumor size with larger size tumors showing the highest degree of ITH. In terms of histologic types, the highest ITH score was observed in tumors with no special type (NST) (mean ± SD of 12.5 ± 25.2) and mixed tumor types (mean ± SD of 11.1 ± 25.9) when compared to special type tumors. In addition, the lowest ITH score was found in invasive lobular carcinoma of the classical type (Supplementary Table S3).

With regard to the levels of ER expression, a higher ITH score was associated with lower ER expression tumors with mean ± SD of 9.4 ± 21.6 compared to 7.1 ± 22.1 in the highest ER-expressing tumors. Similarly, low PR expression tumors had a higher ITH score compared to tumors with high PR expression (*p* < 0.0001). High proliferative tumors (high Ki67 expression) showed a higher ITH score with mean ± SD of 11.9 ± 23.3 compared to 2.0 ± 17.7 in low proliferative tumors (Supplementary Table S3).

Regarding other clinical variables, high ITH scores showed a significant association with younger age, premenopausal status, presence of LVI, and poor NPI scores(*p* < 0.0001) (Table 5).

### 3.4. Outcome Analysis

**Figure 5:**
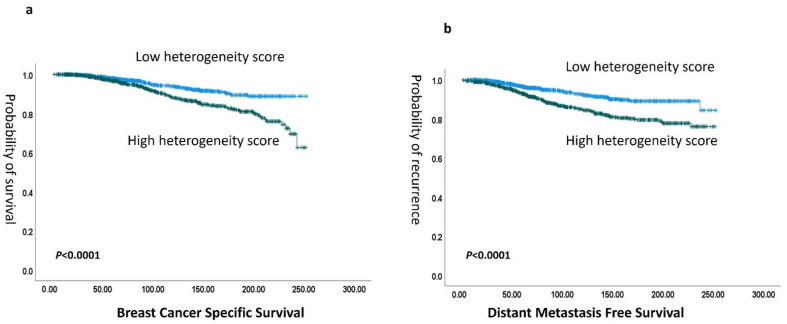
(**a**) Kaplan–Meier association of the association of heterogeneity score with breast cancer-specific survival (BCSS) and (**b**) with distant metastasis-free survival (DMFS).

A high degree of heterogeneity in terms of the individual features showed significant association with shorter patient survival in terms of tumor differentiation heterogeneity (hazard ratio (HR) 3.6 (95% CI: 2.5–4.8; *p* < 0.0001), TIls heterogeneity (HR) 2.5, (95% CI: 1.8–3.6; *p* < 0.001), and stromal heterogeneity (HR) 1.9 (95% CI: 1.4–2.6; *p* < 0.0001)), (Supplementary Figures S2–S4). When the overall ITH score was considered, there was a strong association between high overall ITH score and poor patient survival in terms of shorter BCSS (HR) 1.4 (95% CI: 1.3–1.9; *p* < 0.0001) and DMFS (hazard ratio (HR) 1.1 (95% CI: 1.0–1.4; *p* < 0.0001)) (Figure 5). When stratifying patients according to tumor grade, the ITH score dichotomized grade 2 and/or grade 3 tumors into two distinct prognostic groups (*p* = 0.03 and 0.01, for grade 2 and grade, 3, respectively). Notably, the ITH score significantly categorized patients with moderate NPI risk scores (cohort eligible for Oncotype Dx testing) into two distinct prognostic groups (*p* = 0.04) (Supplementary Figure S1). In a multivariate analysis including the potential confounding factors, tumor grade, LVI, and tumor size, a high ITH score showed an independent association with poor outcomes in terms of BCSS HR 2.2, CI (1.3–3.5), (*p* = 0.001) and DMFS HR 2.3, CI (1.5–3.5), (*p* < 0.0001) (Supplementary Table S4).

**Figure 6:**
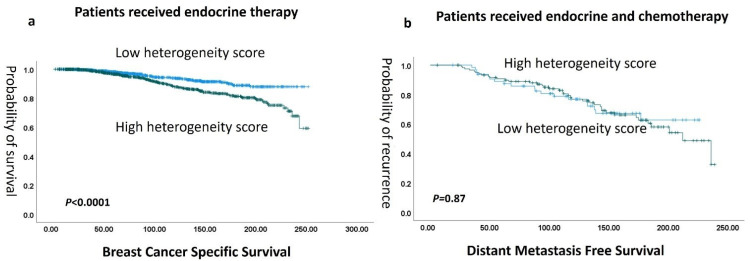
(**a**) Kaplan–Meier association of high heterogeneity score and with shorter BCSS in endocrine therapy treated patients, while no difference in outcome was found in both endocrine and chemotherapy-treated patients (**b**).

In terms of therapy response, there was a significant association between high ITH scores and poor outcomes in patients who received endocrine systemic therapy alone (*p* < 0.0001). However, in the high-risk luminal BC patients who received both endocrine therapy and chemotherapy, ITH lost its association with outcome (*p* = 0.867) (Figure 6)

## 4. Discussion

Breast cancer (BC) exhibits significant intra-tumor genetic diversity, which influences the final morphologic and immunophenotypic features of both tumor cells and their microenvironment [43]. ITH is closely linked to clonal evolution, whereby subclones with a growth advantage expand, whilst less fit subclones are outcompeted and lost [44]. Such tumor heterogeneity plays a crucial role in cancer progression, treatment outcomes, and prognosis [45]. Luminal BC patients are treated with endocrine therapy; however, some patients acquire resistance to targeted therapies and relapse [6]. This resistance may be a direct consequence of pre-existing ITH [38]. However, accurately assessing ITH remains challenging due to its complexity.

Using AI-based tools, it becomes possible to record myriads of morphologic features including spatial architecture and cellular distribution, as well as combine several aspects into one score that can guide patient therapy. AI studies have demonstrated high accuracy in the simultaneous assessment of multiple morphologic features too subtle to be detected with the human eye and which may have the potential to revolutionize routine pathologic practice [24]. AI-based algorithms generate spatial coordinates of features within a tissue context and enable the application of spatial analytics for various ITH aspects [46]. A previous study of kidney cancer generated interpretable features built on the predictions of DL models in the same way as the approach followed in this work, and that study revealed that tumor heterogeneity patterns encode distinct cancer states [47]. Another study showed that deep learning predicted spatial mRNA expression and enabled scalable quantification of intra-tumor heterogeneity to predict patient survival from routine histopathology WSIs in BC [21].

Previous studies have shown that different areas in the same tumor have varying characteristic features [48]. The concepts of ITH and tumor–host interaction within the TME have been used in oncology and pathology to designate many crucial aspects of tumor biology for selecting personalized cancer therapies [49]. Heterogeneity in morphological features, especially histologic tumor grade, is well recognized in routine practice, and the assessment of grade depends on the evaluation of all slides of the tumor to pick the least differentiated areas for assessment of mitoses and nuclear pleomorphism. However, subjective visual quantification of heterogeneity in BC grade is challenging, and this subjectivity decreases the reliability of its use in routine practice [24].

In this study, the power of AI tools has been used to assess and quantify different morphologic features related to ITH in early-stage luminal BC. AI identified and extracted in an unbiased manner features related to the tumor and its associated TME from WSIs using a supervised DL model. To address the challenge of interpreting features from deep learning-based models, the proposed AI pipeline integrates well-established domain-specific features, such as co-occurrence and contrast, to enhance interpretability. Our results showed that most tumors had a degree of heterogeneity with regard to all extracted features as well as the overall ITH score, supporting the concept of ITH even in the luminal class of BC. In this study, several morphologic features showed a high degree of heterogeneity including tumor differentiation, histologic type, tumor cell architecture, cytologic features, tumor-associated stroma, and TILs. This demonstrated the complexity of ITH of BC and suggests a positive correlation between various features in which some tumors show a high degree of ITH incorporating several features, whilst others show minimal variation in the features between different tumor areas. Therefore, analyzing multiple morphologic features can help decipher the morphological complexity of BC and provide a tool to stratify BC patients according to the abundance of these features.

Larger tumor size, grade 3, and NST tumor types showed higher degrees of heterogeneity, including tumor and stromal features, which suggests that ITH may represent the emergence of aggressive tumor clones that drive the poor behavior of these tumors. Moreover, some BC types that are perceived as morphologically and clinically homogeneous show subtle variations within the tumor resulting in high ITH scores and subsequently aggressive behavior. In total, 13% of invasive lobular carcinoma showed high levels of intra-tumor differentiation heterogeneity, which may reflect the existence of more aggressive variants with this tumor type. Also, patients with the highest ER expression showed the lowest degree of ITH compared to patients with low and intermediate ER expression. This may reflect the good response of these patients to endocrine therapy as all tumor cells are similar and respond to therapy, unlike those with low expression, which show a composition of different clones with variable responses.

To further assess ITH in BC, an overall ITH score was developed based on the extracted features, and a high score was detected in about half of the tumors. There was a significant association between higher ITH scores and aggressive tumor characteristics, which provides evidence for the role of ITH in determining tumor behavior.

A significant association was found between high ITH scores and poor patient outcomes, providing further evidence that BC heterogeneity can influence patient prognosis [50]. It has been previously suggested that heterogeneity in luminal BC was associated with poor prognosis [51].

The heterogeneity in grade 2 BC suggests that patients could benefit from more precise stratification. Using the ITH score, these tumors can be divided into two distinct risk levels, each with significant differences in recurrence rates. This approach highlights the importance of personalized treatment plans based on the specific characteristics of the tumor. However, whether tumor heterogeneity influences response to treatment remains to be defined. We found that a higher ITH score predicted poor response to endocrine therapy alone, whilst patients with a low ITH score responded well to endocrine therapy. In contrast, the association between poor outcomes and high ITH was lost when these patients also received chemotherapy. This may be explained by the better response of tumors with high ITH scores to chemotherapy, as these tumors are typically rich in aggressive highly proliferative clones, whereas the low proliferative less aggressive clones responded to endocrine therapy in both groups. This may support offering chemotherapy to tumors with high ITH scores even if they do not appear clinically high risk based on traditional histologic features. However, this assumption needs further investigation in a prospective study. Earlier studies indicated that pre-existing ITH enhances the probability of some tumor cells surviving treatment-induced elimination that could lead to therapy failure [6,12].

This study has some limitations. DCIS segmentation was handled separately from other region segmentation tasks to enhance the focus on DCIS filtering. While this approach simplifies the task, it is feasible to combine DCIS vs. rest segmentation, region segmentation, cell segmentation and classification, and patch classification (digital grade) into a multitask DL model, potentially improving efficiency and performance. Similarly, while nuclei classification plays a significant role in feature calculation, segmentation performance for cells has not been explicitly quantified. Although the quantitative results indicate good performance, selecting an optimal model that balances both classification and segmentation performance would likely improve overall results. Furthermore, our approach separated the texture analysis from the CNN models. While integrating regression into the CNN could enable an end-to-end system, we chose to perform texture analysis independently to retain interpretability and flexibility in feature extraction, particularly for established morphological and textural features. This approach, however, may limit the potential efficiency gains of a fully integrated model. In this study, a cohort of luminal BC was used, and we aim in the future to apply the same algorithm to other cohorts that represent all the molecular classes of BC with the application of the heterogeneity score in a multicentric study to further evaluate its clinical value in different molecular classes.

## 5. Conclusions

The results of this study indicate that AI-based tools can be used to assess morphological features representative of ITH in BC. ITH provides independent prognostic information in early-stage luminal BC that can be used to guide therapy decision making.

## Figures and Tables

**Table 1:** Training and validation of DL models.

Module	Metric			Subset	ROI Size
DCIS filter (CNN_DCIS_)	F1-score			5-fold cross-validation on annotated tiles (*n* = 13,981) of size 1024 × 1024 pixels extracted from ROIs in the discovery set	Variable size box drawn at 5× magnification to cover a visual field
	Tumor	DCIS			
	0.71 ± 0.03	0.90 ± 0.01			
Region segmentation (CNN_Reg_)	Dice coefficient			Holdout validation (trained on annotated ROIs (*n* = 193) [3] in the discovery set, validated on ROIs (*n* = 48) in the internal validation set)	Variable size box drawn at 5× magnification to cover a visual field
	Stroma	Other			
	0.76	0.69			
Nuclei classification (CNN_Nuc_)	F1-score			3-fold cross-validation on ROIs (*n* = 83) [3] in the discovery set	Variable size box drawn at 20× magnification to cover a visual field
	Immune	Tumor	Connective		
	0.82 ± 0.06	0.92 ± 0.02	0.81 ± 0.03		
Digital grade prediction (CNN_DG_)	ROC-AUC			3-fold cross-validation on ROIs (1 per WSI) from discovery set	5600 × 5600 pixels at 40× magnification
	0.83 ± 0.01				

ROC-AUC for predicting digital grades was derived from a linear SVM trained using the proportions of local grades in each whole slide image (WSI), with clinical grade serving as the ground truth. For feature extraction, all models were applied to the entire slide rather than specific regions of interest (ROIs). The values are presented as the mean ± standard deviation (x ± SD) to indicate one standard deviation from the mean.

**Table 2:** 3-fold cross-validation results of nuclei classification.

Model				Overall (Macro-Average)								
				Precision			Recall			F1		
M1				0.69 ± 0.05			0.52 ± 0.08			0.55 ± 0.08		
M2				0.66 ± 0.04			0.62 ± 0.07			0.61 ± 0.05		
M3				0.68 ± 0.01			0.68 ± 0.01			0.62 ± 0.01		
M4				0.69 ± 0.02			0.66 ± 0.01			0.62 ± 0.02		
CNN_Nuc_				0.82 ± 0.09			0.79 ± 0.02			0.79 ± 0.07		
**Cell-Wise Classification Results**												
**Model**	**Tumor**			**Immune**			**Connective**			**Normal epithelial**		
	**Pr**	**Re**	**F1**	**Pr**	**Re**	**F1**	**Pr**	**Re**	**F1**	**Pr**	**Re**	**F1**
M1	0.75± 0.02	0.95 ± 0.02	0.83 ± 0.01	0.94 ± 0.03	0.57 ± 0.11	0.71 ± 0.07	0.88 ± 0.06	0.32 ± 0.06	0.47 ± 0.07	0.18 ± 0.19	0.24 ± 0.17	0.20 ± 0.18
M2	0.91 ± 0.03	0.81± 0.02	0.87 ± 0.01	0.84 ± 0.04	0.64 ± 0.12	0.72 ± 0.10	0.82 ± 0.07	0.63 ± 0.04	0.71 ± 0.04	0.08± 0.06	0.40 ± 0.24	0.13 ± 0.08
M3	0.92 ± 0.03	0.71 ± 0.09	0.80 ± 0.07	0.89 ± 0.05	0.67 ± 0.07	0.77 ± 0.04	0.81 ± 0.04	0.65 ± 0.04	0.73 ± 0.04	0.10 ± 0.03	0.69 ± 0.05	0.18 ± 0.06
M4	0.91 ± 0.03	0.81 ± 0.01	0.85 ± 0.02	0.91 ± 0.03	0.64 ± 0.09	0.75 ± 0.06	0.83 ± 0.08	0.63 ± 0.05	0.71 ± 0.01	0.11 ± 0.04	0.55 ± 0.08	0.18 ± 0.05
CNN_Nuc_	0.92 ± 0.01	0.92 ± 0.04	0.92 ± 0.02	0.92 ± 0.04	0.74 ± 0.11	0.82 ± 0.06	0.81 ± 0.04	0.81 ± 0.03	0.81 ± 0.03	0.60 ± 0.33	0.69 ± 0.08	0.59 ± 0.22

M1: HoVer-Net pretrained on PanNuke-Breast dataset, M2: Inception ResNet v2, M3: SC-CNN, M4: Inception V3. M2-4 are fine-tuned on target dataset. Pr: precision, Re: recall. x ± sd for precision, recall and F1 represents one standard deviation of the mean (mean ± standard) across 3-fold.

**Table 3:** Relationship between tumoral differentiation features of intra-tumor heterogeneity and clinicopathological parameters in luminal BC.

Variables	Tumor Differentiation Heterogeneity		X^2^ *p*-Value
	Low (n, %)	High (n, %)	
**Age** <50 ≥50	314 (52) 1289 (67)	291 (48) 634 (33)	45.4 **<0.0001**
**Menopausal state** Premenopausal Post-menopausal	357 (54) 1270 (67)	302 (46) 435 (33)	33.5 **<0.0001**
**Tumor size** ≤2 cm >2 cm	1312 (70) 314 (46)	564 (30) 386 (54)	123.3 **<0.0001**
**Tumor grade** Grade 1 Grade 2 Grade 3	543 (90) 980 (68) 104 (20)	40 (10) 458 (32) 415 (80)	618.2 **<0.0001**
**Histologic tumor types** No special type (NST) Lobular Other special types Mixed subtype	738 (52) 286 (85) 154 (92) 449 (71)	690 (48) 50 (15) 14 (8) 180 (29)	228.3 **<0.0001**
**Lymph node status** Negative Positive	1299 (67) 328 (52)	638 (33) 300 (48)	45.9 **<0.0001**
**Lymphovascular invasion** Absent Present	1476 (68) 151 (38)	688 (32) 246 (62)	131.8 **<0.0001**
**Nottingham prognostic index** Good prognostic group Moderate prognostic group Poor prognostic group	1144 (80) 466 (45) 17 (17)	286 (20) 564 (55) 86 (83)	416.4 **<0.0001**
**ER expression levels.** Lower < 10% Intermediate (10–99%) Highest (100%)	6 (22) 417 (57) 1196 (67)	21 (78) 311 (43) 592 (33)	40.9 **<0.0001**
**PR status** Low ≤ 10% High > 10%	305 (61) 1144 (63)	193 (39) 667 (37)	0.62 0.43
**Ki67 expression** Low ≤ 10 High > 10	814 (81) 694 (50)	194 (19) 694 (50)	236.8 **<0.0001**

ER, estrogen receptor. PR, Progesterone receptor. Significant *p* values are in bold.

**Table 4:** Relationship between stromal and tumor-infiltrating lymphocytes (ITH) features of intra-tumor heterogeneity and clinicopathological parameters in luminal breast cancer.

Variables	Stromal Heterogeneity		X^2^ *p*-Value	TILS Heterogeneity		X^2^ *p*-Value
	Low (n, %)	High (n, %)		Low (n, %)	High (n, %)	
**Age (years)** <50 ≥50	290 (48) 1208 (63)	315 (52) 714 (35)	42.4 **<0.0001**	487 (80) 1674 (87)	118 (20) 248 (13)	16.19 **<0.0001**
**Menopausal state** Premenopausal Post-menopausal	322 (49) 1188 (63)	337 (51) 713 (37)	37.6 **<0.0001**	531 (81) 1657 (87)	128 (19) 244 (13)	17.1 **<0.0001**
**Tumor size** ≤2 cm >2 cm	1288 (69) 222 (33)	588 (31) 459 (67)	268.6 **<0.0001**	1752 (93) 435 (64)	123 (7) 247 (36)	355.4 **<0.0001**
**Tumor grade** Grade 1 Grade 2 Grade 3	439 (73) 868 (62) 203 (39)	165 (27) 570 (40) 315 (61)	131.9 **<0.0001**	393 (95) 909 (90) 253 (72)	21 (5) 101 (10) 96 (27)	99.8 **<0.0001**
**Histologic tumor types** No special type (NST) Lobular Other special types Mixed subtype	765 (54) 219 (65) 131 (78) 395 (63)	663 (46) 117 (35) 37 (22) 37 (22)	51.6 **<0.0001**	1179 (83) 289 (86) 160 (95) 560 (89)	248 (17) 47 (14) 8 (5) 69 (11)	28.7 **<0.0001**
**Lymph node status** Negative Positive	1210 (67) 300 (48)	722 (33) 328 (52)	43.2 **<0.0001**	1701 (88) 487 (77)	231 (12) 141 (23)	42.03 **<0.0001**
**Lymphovascular invasion** Absent Present	1334 (62) 176 (44)	829 (38) 221 (56)	41.7 **<0.0001**	1900 (88) 288 (73)	263 (12) 109 (27)	63.19 **<0.0001**
**Nottingham prognostic index** Good prognostic group Moderate prognostic group Poor prognostic group	1019 (71) 469 (46) 22 (21)	409 (29) 560 (54) 81 (79)	227.1 **<0.0001**	1159 (95) 780 (76) 49 (47)	68 (5) 250 (24) 54 (53)	307.4 **<0.0001**
**ER expression levels** Lower < 10% Intermediate (10–99%) Highest (100%)	19 (70) 425 (58) 1057 (59)	8 (30) 303 (42) 730 (41)	1.57 0.46	20 (74) 609 (84) 1545 (86)	7 (26) 118 (16) 243 (14)	5.8 0.05
**PR status** Low ≤ 10% High > 10%	298 (60) 1052 (58)	200 (40) 758 (42)	0.47 0.49	413 (83) 1560 (86)	85 (17) 250 (14)	3.3 0.06
**Ki67 expression** Low ≤ 10 High > 10	724 (72) 683 (49)	284 (28) 704 (51)	122.8 **<0.0001**	949 (94) 1101 (79)	59 (6) 286 (21)	103.2 **<0.0001**

ER, estrogen receptor. PR, progesterone receptor. Significant *p* values are in bold.

**Table 5:** Relationship between overall heterogeneity score and clinicopathological parameters in the study cohort.

Variables	Heterogeneity Score		X^2^ *p*-Value
	Low (n, %)	High (n, %)	
**Age** <50 ≥50	396 (66) 452 (52)	208 (34) 411 (48)	7.7 **0.005**
**Menopausal state** Premenopausal Post-menopausal	312 (47) 1102 (58)	347 (53) 800 (42)	22.2 **<0.0001**
**Tumor size** ≤2 cm >2 cm	882 (64) 157(40)	498 (36) 237 (60)	73.1 **<0.0001**
**Tumor grade** Grade 1 Grade 2 Grade 3	396 (66) 814 (57) 204 (39)	208 (34) 624 (43) 315 (61)	80.4 **<0.0001**
**Histologic tumor types** Non-special type (NST) Lobular Other special types NST mixed	716 (51) 240 (71) 126 (75) 332 (53)	712 (49) 96 (29) 42 (25) 297 (47)	78.7 **<0.0001**
**Lymph node status** Absent Present	11,145 (59) 269 (43)	788 (41) 359 (57)	51.6 **<0.0001**
**Lymphovascular invasion** Absent Present	1258 (58) 156 (39)	906 (42) 241 (61)	48.1 **<0.0001**
**Nottingham prognostic index** Good prognostic group Moderate prognostic group Poor prognostic group	932 (65) 461 (45) 21 (20)	496 (35) 569 (55) 82 (80)	154.4 **<0.0001**
**ER expression levels** Lower < 10% Intermediate (10–99%) Highest (100%)	10 (62) 263 (56) 762 (60)	6 (38) 207 (44) 518 (40)	1.9 0.38
**PR status** Low ≤ 10% High > 10%	316 (64) 944 (52)	182 (36) 867 (48)	20.2 **<0.0001**
**Ki67 expression** Low ≤ 10% High > 10%	683 (68) 633 (46)	325 (32) 755 (54)	115.7 **<0.0001**

ER, estrogen receptor. PR, progesterone receptor. Significant *p* values are in bold.

## Data Availability

All data used in this study are archived and could be available on a reasonable request.

## Supplementary Materials

The following supporting information can be downloaded at: https://www.mdpi.com/article/10.3390/cancers16223849/s1, Figure S1: shows Kaplan Meier association of heterogeneity score with breast cancer-specific in intermediate risk patients (moderate Nottingham prognostic index); Figure S2: shows Kaplan Meier association of tumour differentiation heterogeneity (high vs low) with breast cancer-specific survival; Figure S3: shows Kaplan Meier association of stromal heterogeneity (high vs low) with breast cancer-specific survival; Figure S4: shows Kaplan Meier association of tumour infiltrating lymphocytes (TILS) heterogeneity (high vs low) with breast cancer-specific survival; Table S1: Clinicopathological characteristics of the study cohort; Table S2: overview of texture features employed in current study; Table S3: Mean and standard deviation (Mean ± SD) of heterogeneity score in the different pathological parameters; Table S4: Multivariate analysis of overall intra-tumour heterogeneity score in luminal breast cancer with prognostic parameters.

## References

[1] Fumagalli C., Barberis M. (2021). Breast Cancer Heterogeneity. Diagnostics.

[2] Marusyk A., Polyak K. (2010). Tumor heterogeneity: Causes and consequences. Biochim. Et Biophys. Acta.

[3] McQuerry J.A., Chang J.T., Bowtell D.D.L., Cohen A., Bild A.H. (2017). Mechanisms and clinical implications of tumor heterogeneity and convergence on recurrent phenotypes. J. Mol. Med..

[4] Malhotra G.K., Zhao X., Band H., Band V. (2010). Histological, molecular and functional subtypes of breast cancers. Cancer Biol. Ther..

[5] Stanta G., Bonin S. (2018). Overview on Clinical Relevance of Intra-Tumor Heterogeneity. Front. Med..

[6] Marusyk A., Janiszewska M., Polyak K. (2020). Intratumor Heterogeneity: The Rosetta Stone of Therapy Resistance. Cancer Cell.

[7] Gerlinger M., Rowan A.J., Horswell S., Larkin J., Endesfelder D., Gronroos E., Martinez P., Matthews N., Stewart A., Tarpey P. (2012). Intratumor Heterogeneity and Branched Evolution Revealed by Multiregion Sequencing. N. Engl. J. Med..

[8] Zhou Y., Tao L., Qiu J., Xu J., Yang X., Zhang Y., Tian X., Guan X., Cen X., Zhao Y. (2024). Tumor biomarkers for diagnosis, prognosis and targeted therapy. Signal Transduct. Target. Ther..

[9] Aleskandarany M.A., Green A.R., Ashankyty I., Elmouna A., Diez-Rodriguez M., Nolan C.C., Ellis I.O., Rakha E.A. (2016). Impact of intratumoural heterogeneity on the assessment of Ki67 expression in breast cancer. Breast Cancer Res. Treat..

[10] Lloyd M.C., Alfarouk K.O., Verduzco D., Bui M.M., Gillies R.J., Ibrahim M.E., Brown J.S., Gatenby R.A. (2014). Vascular measurements correlate with estrogen receptor status. BMC Cancer.

[11] Zilenaite D., Rasmusson A., Augulis R., Besusparis J., Laurinaviciene A., Plancoulaine B., Ostapenko V., Laurinavicius A. (2020). Independent Prognostic Value of Intratumoral Heterogeneity and Immune Response Features by Automated Digital Immunohistochemistry Analysis in Early Hormone Receptor-Positive Breast Carcinoma. Front. Oncol..

[12] Laurinavicius A., Plancoulaine B., Rasmusson A., Besusparis J., Augulis R., Meskauskas R., Herlin P., Laurinaviciene A., Abdelhadi Muftah A.A., Miligy I. (2016). Bimodality of intratumor Ki67 expression is an independent prognostic factor of overall survival in patients with invasive breast carcinoma. Virchows Arch. Int. J. Pathol..

[13] Althobiti M., Aleskandarany M.A., Joseph C., Toss M., Mongan N., Diez-Rodriguez M., Nolan C.C., Ashankyty I., Ellis I.O., Green A.R. (2018). Heterogeneity of tumour-infiltrating lymphocytes in breast cancer and its prognostic significance. Histopathology.

[14] Beca F., Polyak K. (2016). Intratumor Heterogeneity in Breast Cancer. Adv. Exp. Med. Biol..

[15] Joseph C., Papadaki A., Althobiti M., Alsaleem M., Aleskandarany M.A., Rakha E.A. (2018). Breast cancer intratumour heterogeneity: Current status and clinical implications. Histopathology.

[16] Lashen A., Al-Kawaz A., Jeyapalan J.N., Alqahtani S., Shoqafi A., Algethami M., Toss M., Green A.R., Mongan N.P., Sharma S. (2024). Immune infiltration, aggressive pathology, and poor survival outcomes in RECQL helicase deficient breast cancers. Neoplasia.

[17] Makki J. (2015). Diversity of Breast Carcinoma: Histological Subtypes and Clinical Relevance. Clin. Med. Insights Pathol..

[18] Richard V., Nair M.G., Jaikumar V.S., Jones S., Prabhu J.S., Kerin M.J. (2023). Cell State Transitions and Phenotypic Heterogeneity in Luminal Breast Cancer Implicating MicroRNAs as Potential Regulators. Int. J. Mol. Sci..

[19] Acs B., Rantalainen M., Hartman J. (2020). Artificial intelligence as the next step towards precision pathology. J. Intern. Med..

[20] Wahab N., Toss M., Miligy I.M., Jahanifar M., Atallah N.M., Lu W., Graham S., Bilal M., Bhalerao A., Lashen A.G. (2023). AI-enabled routine H&E image based prognostic marker for early-stage luminal breast cancer. NPJ Precis. Oncol..

[21] Wang Y., Ali M.A., Vallon-Christersson J., Humphreys K., Hartman J., Rantalainen M. (2023). Transcriptional intra-tumour heterogeneity predicted by deep learning in routine breast histopathology slides provides independent prognostic information. Eur. J. Cancer.

[22] AbdulJabbar K., Raza S.E.A., Rosenthal R., Jamal-Hanjani M., Veeriah S., Akarca A., Lund T., Moore D.A., Salgado R., Al Bakir M. (2020). Geospatial immune variability illuminates differential evolution of lung adenocarcinoma. Nat. Med..

[23] Plancoulaine B., Laurinaviciene A., Herlin P., Besusparis J., Meskauskas R., Baltrusaityte I., Iqbal Y., Laurinavicius A. (2015). A methodology for comprehensive breast cancer Ki67 labeling index with intra-tumor heterogeneity appraisal based on hexagonal tiling of digital image analysis data. Virchows Arch..

[24] Laurinavicius A., Rasmusson A., Plancoulaine B., Shribak M., Levenson R. (2021). Machine-Learning–Based Evaluation of Intratumoral Heterogeneity and Tumor-Stroma Interface for Clinical Guidance. Am. J. Pathol..

[25] Lashen A., Toss M.S., Green A.R., Mongan N.P., Rakha E. (2022). Ki67 assessment in invasive luminal breast cancer: A comparative study between different scoring methods. Histopathology.

[26] Lashen A.G., Toss M.S., Mongan N.P., Green A.R., Rakha E.A. (2023). The clinical value of progesterone receptor expression in luminal breast cancer: A study of a large cohort with long-term follow-up. Cancer.

[27] Lashen A.G., Toss M.S., Katayama A., Gogna R., Mongan N.P., Rakha E.A. (2021). Assessment of proliferation in breast cancer: Cell cycle or mitosis? An observational study. Histopathology.

[28] Wahab N., Miligy I.M., Dodd K., Sahota H., Toss M., Lu W., Jahanifar M., Bilal M., Graham S., Park Y. (2022). Semantic annotation for computational pathology: Multidisciplinary experience and best practice recommendations. J. Pathol. Clin. Res..

[29] Makhlouf S., Wahab N., Toss M., Ibrahim A., Lashen A.G., Atallah N.M., Ghannam S., Jahanifar M., Lu W., Graham S. (2023). Evaluation of tumour infiltrating lymphocytes in luminal breast cancer using artificial intelligence. Br. J. Cancer.

[30] Szegedy C., Vanhoucke V., Ioffe S., Shlens J., Wojna Z. Rethinking the Inception Architecture for Computer Vision. Proceedings of the 2016 IEEE Conference on Computer Vision and Pattern Recognition (CVPR).

[31] Russakovsky O., Deng J., Su H., Krause J., Satheesh S., Ma S., Huang Z., Karpathy A., Khosla A., Bernstein M. (2015). ImageNet Large Scale Visual Recognition Challenge. Int. J. Comput. Vis..

[32] Wang C., Zhao H. (2018). Spatial Heterogeneity Analysis: Introducing a New Form of Spatial Entropy. Entropy.

[33] van der Walt S., Schönberger J.L., Nunez-Iglesias J., Boulogne F., Warner J.D., Yager N., Gouillart E., Yu T. (2014). The scikit-image contributors. 2014. Scikit-image: Image processing in Python. PeerJ.

[34] Davnall F., Yip C.S., Ljungqvist G., Selmi M., Ng F., Sanghera B., Ganeshan B., Miles K.A., Cook G.J., Goh V. (2012). Assessment of tumor heterogeneity: An emerging imaging tool for clinical practice?. Insights Imaging.

[35] Azimi V., Chang Y.H., Thibault G., Smith J., Tsujikawa T., Kukull B., Jensen B., Corless C., Margolin A., Gray J.W. (2017). Breast cancer histopathology image analysis pipeline for tumor purity estimation. Proc. IEEE Int. Symp. Biomed. Imaging.

[36] Lawson D.J., Solanki V., Yanovich I., Dellert J., Ruck D., Endicott P. (2021). CLARITY: Comparing heterogeneous data using dissimilarity. R. Soc. Open Sci..

[37] Higgins J.P., Thompson S.G., Deeks J.J., Altman D.G. (2003). Measuring inconsistency in meta-analyses. BMJ (Clin. Res. Ed.).

[38] Nunes A., Trappenberg T., Alda M. (2020). The definition and measurement of heterogeneity. Transl. Psychiatry.

[39] Stephen Gilliver N.V. (2016). How to interpret and report the results from multivariable analyses. Med. Writ..

[40] Camp R.L., Dolled-Filhart M., Rimm D.L. (2004). X-tile: A new bio-informatics tool for biomarker assessment and outcome-based cut-point optimization. Clin. Cancer Res. Off. J. Am. Assoc. Cancer Res..

[41] Lashen A., Toss M.S., Fadhil W., Oni G., Madhusudan S., Rakha E. (2023). Evaluation Oncotype DX^®^ 21-Gene Recurrence Score and Clinicopathological Parameters: A single institutional experience. Histopathology.

[42] Makhlouf S., Quinn C., Toss M., Alsaleem M., Atallah N.M., Ibrahim A., Rutland C.S., Mongan N.P., Rakha E.A. (2024). Quantitative expression of oestrogen receptor in breast cancer: Clinical and molecular significance. Eur. J. Cancer.

[43] Lüönd F., Tiede S., Christofori G. (2021). Breast cancer as an example of tumour heterogeneity and tumour cell plasticity during malignant progression. Br. J. Cancer.

[44] Greaves M., Maley C.C. (2012). Clonal evolution in cancer. Nature.

[45] Fisher R., Pusztai L., Swanton C. (2013). Cancer heterogeneity: Implications for targeted therapeutics. Br. J. Cancer.

[46] Heindl A., Nawaz S., Yuan Y. (2015). Mapping spatial heterogeneity in the tumor microenvironment: A new era for digital pathology. Lab. Investig..

[47] Nyman J., Denize T., Bakouny Z., Labaki C., Titchen B.M., Bi K., Hari S.N., Rosenthal J., Mehta N., Jiang B. (2023). Spatially aware deep learning reveals tumor heterogeneity patterns that encode distinct kidney cancer states. bioRxiv.

[48] He L., Long L.R., Antani S., Thoma G.R. (2012). Histology image analysis for carcinoma detection and grading. Comput. Methods Programs Biomed..

[49] Ramón Y.C.S., Sesé M., Capdevila C., Aasen T., De Mattos-Arruda L., Diaz-Cano S.J., Hernández-Losa J., Castellví J. (2020). Clinical implications of intratumor heterogeneity: Challenges and opportunities. J. Mol. Med..

[50] Turashvili G., Brogi E. (2017). Tumor Heterogeneity in Breast Cancer. Front. Med..

[51] Ma D., Jiang Y.-Z., Liu X.-Y., Liu Y.-R., Shao Z.-M. (2017). Clinical and molecular relevance of mutant-allele tumor heterogeneity in breast cancer. Breast Cancer Res. Treat..

