# Supplementary material for: Characterization of Breast Cancer Intra-Tumor Heterogeneity Using Artificial Intelligence
Source: Cancers (Basel). 2024 Nov 16;16(22):3849. doi: 10.3390/cancers16223849 (PMC11593220; doi:10.3390/cancers16223849)
Supplement: Supplementary file 1 [file cancers-16-03849-s001.zip › cancers-3201412-supplementary.pdf]

SUPPLEMENTARY MATERIALS

Supplementary table (1) Clinicopathological characteristics of the study cohort.

Variables	Number (%)
**Age (years)**	
<50	605 (25)
≥50	1923 (75)
**Menopausal state**	
Premenopausal	659 (26)
Post-menopausal	1902 (74)
**Tumour size (cm)**	
≤2	1876 (73)
>2	682 (27)
**Histologic tumour grade**	
Grade 1	604 (24)
Grade 2	1438 (56)
Grade 3	519 (20)
**Histologic tumour types**	
No Special Type (NST)	1428 (56)
Lobular	336 (13)
Other special types	168 (7)
Mixed subtypes	629 (24)
**Lymph node status**	
Negative	1933 (76)
Positive	628 (24)
**Lymhovascular invasion**	
Absent	2164 (85)
Present	397 (15)
**Nottingham prognostic index**	
Good prognostic group	1428 (56)
Moderate prognostic group	1030 (40)
Poor prognostic group	103 (4)
**ER expression levels.**	
Lower <10%	27 (2)
Intermediate (10-99%)	728 (28)
Highest (100%)	1788 (70)
**PR status**	
Negative ≤10%	498 (22)
Positive >10%	1811 (78)
**Ki67 expression**	
Low ≤10%	1008 (43)
High >10%	1388 (57)

ER: estrogen receptor, PR Progesterone receptor

Supplementary table (2): overview of texture features employed in current study

No	Intra-tumour heterogeneity extracted features	
	**Feature**	**Description**
1	g1g1_cooccur	Number of times a grade1 predicted patch (size 62 micro m) cooccur with grade1 patch over the whole image.
2	g1g2_cooccur	Number of times a grade1 predicted patch (size 62 micro m) cooccur with grade2 patch.
3	g1g3_cooccur	Number of times a grade1 predicted patch (size 62 micro m) cooccur with grade3 patch.
4	g2g3_cooccur	Number of times a grade2 predicted patch (size 123 micro m) cooccur with grade3 patch.
5	g-ASM	ASM from the cooccurrence matrix of the grade predictions for patches of size 123 micro m.
6	g-contrast	Contrast from the cooccurrence matrix of the grade predictions for patches of size 123 micro m.
7	g-correlation	Correlation from the cooccurrence matrix of the grade predictions for patches of size 123 micro m.
8	g -dissimilarity	Dissimilarity from the cooccurrence matrix of the grade predictions for patches of size 123 micro m.
9	g-energy	Energy from the cooccurrence matrix of the grade predictions for patches of size 123 micro m.
10	g-heterogeneity	Heterogeneity from the cooccurrence matrix of the grade predictions for patches of size 123 micro m.
11	p1p2_cooccur	Number of times a pleomorphic1 predicted patch (size 123 micro m) cooccur with pleomorphic2 patch.
12	p1p3_cooccur	Number of times a pleomorphic1 predicted patch (size 123 micro m) cooccur with pleomorphic3 patch.
13	p2p3_cooccur	Number of times a pleomorphic2 predicted patch (size 123 micro m) cooccur with pleomorphic3 patch.
14	p-ASM	ASM from the cooccurrence matrix of the pleomorphic predictions for patches of size 123 micro m.
15	p-contrast	Contrast from the cooccurrence matrix of the pleomorphic predictions for patches of size 123 micro m.
16	p-correlation	Correlation from the cooccurrence matrix of the pleomorphic predictions for patches of size 123 micro m.
17	p-dissimilarity	Dissimilarity from the cooccurrence matrix of the pleomorphic predictions for patches of size 123 micro m.
18	p-energy	Energy from the cooccurrence matrix of the pleomorphic predictions for patches of size 123 micro m.
19	p-heterogeneity	Heterogeneity from the cooccurrence matrix of the pleomorphic predictions for patches of size 123 micro m.
20	TILs heterogeneity	Heterogeneity from the cooccurrence matrix of the number of lymphocytes predicted for patches of size 62 micro m.? whole slide
21	TILs dissimilarity	Dissimilarity from the cooccurrence matrix of the number of lymphocytes predicted for patches of size 62 micro m.
22	TILs contrast	Contrast from the cooccurrence matrix of the number of lymphocytes predicted for patches of size 62 micro m.
23	TILs correlation	Correlation from the cooccurrence matrix of the number of lymphocytes predicted for patches of size 62 micro m.
24	TILs energy	Energy from the cooccurrence matrix of the number of lymphocytes predicted for patches of size 62 micro m.
25	TILs ASM	ASM from the cooccurrence matrix of the number of lymphocytes predicted for patches of size 62 micro m.
26	Stromal heterogeneity	Heterogeneity from the cooccurrence matrix of the number of lymphocytes predicted for patches of size 62 micro m.? stroma or tills and in while slide.
27	Stromal dissimilarity	Dissimilarity from the cooccurrence matrix of the number of lymphocytes predicted for patches of size 62 micro m.
28	Stromal contrast	Contrast from the cooccurrence matrix of the number of lymphocytes predicted for patches of size 62 micro m.
29	Stromal correlation	correlation from the cooccurrence matrix of the number of lymphocytes predicted for patches of size 62 micro m.
30	Stromal energy	Energy from the cooccurrence matrix of the number of lymphocytes predicted for patches of size 62 micro m.
31	Stromal ASM	ASM from the cooccurrence matrix of the number of lymphocytes predicted for patches of size 62 micro m.
32	G1g2_patches_all	The number of windows (size 369 micro m) where grade1 and grade2 patches (size 123 micro m) cooccurred.
33	G1g1_patches_high	The number of windows (size 369 micro m) where grade1 and grade1 patches (size 123 micro m) cooccurred above a threshold of **4.84** (calculated from the discovery cohort of about 550).
34	G1g1_patches_low	Number of windows (size 369 micro m) where grade1 and grade1 patches (size 123 micro m) cooccurred below a threshold of 4.84 (calculated from the discovery cohort of about 550).
35	G1g2_patches_high	Number of windows (size 369 micro m) where grade1 and grade2 patches (size 123 micro m) cooccurred above a threshold of 2.28 (calculated from the discovery cohort of about 550).
36	G1g2_patches_low	The number of windows (size 369 micro m) where grade1 and grade2 patches (size 123 micro m) cooccurred below a threshold of 2.28 (calculated from the discovery cohort of about 550).
37	G1g3_patches_high	The number of windows (size 369 micro m) where grade1 and grade3 patches (size 123 micro m) cooccurred above a threshold of 3.16 (calculated from the discovery cohort of about 550).
38	G1g3_patches_low	The number of windows (size 369 micro m) where grade1 and grade3 patches (size 123 micro m) cooccurred below a threshold of 3.16 (calculated from the discovery cohort of about 550).
39	G2g2_patches_high	Number of windows (size 369 micro m) where grade2 and grade2 patches (size 123 micro m) cooccurred above a threshold of 0.84 (calculated from the discovery cohort of about 550).
40	G2g2_patches_low	The number of windows (size 369 micro m) where grade2 and grade2 patches (size 123 micro m) cooccurred below a threshold of 0.84 (calculated from the discovery cohort of about 550).

41	G2g3_patches_high	Number of windows (size 369 micro m) where grade2 and grade3 patches (size 123 micro m) cooccurred above a threshold of 1.49 (calculated from the discovery cohort of about 550).
42	G2g3_patches_low	Number of windows (size 369 micro m) where grade2 and grade3 patches (size 123 micro m) cooccurred below a threshold of 1.49 (calculated from the discovery cohort of about 550).
43	G3g3_patches_high	Number of windows (size 369 micro m) where grade3 and grade3 patches (size 123 micro m) cooccurred above a threshold of 2.54 (calculated from the discovery cohort of about 550).
44	G3g3_patches_low	The number of windows (size 369 micro m) where grade3 and grade3 patches (size 123 micro m) cooccurred below a threshold of 2.54 (calculated from the discovery cohort of about 550).
45	p1p1_patches_high	Number of windows (size 369 micro m) where pleo1 and pleo1 patches (size 123 micro m) cooccurred above a threshold of 0.61 (calculated from the discovery cohort of about 550).
46	p1p1_patches_low	The number of windows (size 369 micro m) where pleo1 and pleo1 patches (size 123 micro m) cooccurred below a threshold of 0.61 (calculated from the discovery cohort of about 550).
47	p1p2_patches_high	The number of windows (size 369 micro m) where pleo1 and pleo2 patches (size 123 micro m) cooccurred above a threshold of 1.66 (calculated from the discovery cohort of about 550).
48	p1p2_patches_low	Number of windows (size 369 micro m) where pleo1 and pleo2 patches (size 123 micro m) cooccurred below a threshold of 1.66 (calculated from the discovery cohort of about 550).
49	p1p3_patches_high	The number of windows (size 369 micro m) where pleo1 and pleo3 patches (size 123 micro m) cooccurred above a threshold of 1.30 (calculated from the discovery cohort of about 550).
50	p1p3_patches_low	The number of windows (size 369 micro m) where pleo1 and pleo3 patches (size 123 micro m) cooccurred below a threshold of 1.30 (calculated from the discovery cohort of about 550).
51	p2p2_patches_high	Number of windows (size 369 micro m) where pleo2 and pleo2 patches (size 123 micro m) cooccurred above a threshold of 3.45 (calculated from the discovery cohort of about 550).
52	p2p2_patches_low	Number of windows (size 369 micro m) where pleo2 and pleo2 patches (size 123 micro m) cooccurred below a threshold of 3.45 (calculated from the discovery cohort of about 550).
53	p2p3_patches_high	The number of windows (size 369 micro m) where pleo2 and pleo3 patches (size 123 micro m) cooccurred above a threshold of 3.61 (calculated from the discovery cohort of about 550).
54	p2p3_patches_low	The number of windows (size 369 micro m) where pleo2 and pleo3 patches (size 123 micro m) cooccurred below a threshold of 3.61 (calculated from the discovery cohort of about 550).
55	p3p3_patches_high	The number of windows (size 369 micro m) where pleo3 and pleo3 patches (size 123 micro m) cooccurred above a threshold of 4.17 (calculated from the discovery cohort of about 550).
56	p3p3_patches_low	The number of windows (size 369 micro m) where pleo3 and pleo3 patches (size 123 micro m) cooccurred below a threshold of 4.17 (calculated from the discovery cohort of about 550).
57	G1g2_cooccur_score	G1g2_cooccur/(G1g1_cooccur+ G1g2_cooccur+ G1g2_cooccur).
58	G1g3_cooccur_score	G1g3_cooccur/(G1g1_cooccur+ G1g2_cooccur+ G1g3_cooccur).
59	G2g3_cooccur_score	G2g3_cooccur/(G1g2_cooccur+ G2g2_cooccur+ G2g3_cooccur).
60	Inter_WSI_g1g1_patch_cooccur_high	Cooccurrence of grade1 and grade1 patches (size 123 micro m) above a threshold in windows (size 369 micro m). cooccurrence threshold of 4.84 (calculated from the discovery cohort of about 550).
61	Inter_WSI_g1g1_patch_cooccur_low	Cooccurrence of grade1 and grade1 patches (size 123 micro m) below a threshold in windows (size 369 micro m). cooccurrence threshold of 4.84 (calculated from the discovery cohort of about 550).
62	Inter_WSI_g1g2_patch_cooccur_high	Cooccurrence of grade1 and grade2 patches (size 123 micro m) above a threshold in windows (size 369 micro m). cooccurrence threshold of 2.28 (calculated from the discovery cohort of about 550).
63	Inter_WSI_g1g2_patch_cooccur_low	Cooccurrence of grade1 and grade2 patches (size 123 micro m) below a threshold in windows (size 369 micro m). cooccurrence threshold of 2.28 (calculated from the discovery cohort of about 550).
64	Inter_WSI_g1g3_patch_cooccur_high	Cooccurrence of grade1 and grade3 patches (size 123 micro m) above a threshold in windows (size 369 micro m). cooccurrence threshold of 3.16 (calculated from the discovery cohort of about 550).
65	Inter_WSI_g1g3_patch_cooccur_low	Cooccurrence of grade1 and grade3 patches (size 123 micro m) below a threshold in windows (size 369 micro m). cooccurrence threshold of 3.16 (calculated from the discovery cohort of about 550).
66	Inter_WSI_g2g2_patch_cooccur_high	Cooccurrence of grade 2 and grade 2 patches (size 123 micro m) above a threshold in windows (size 369 micro m). cooccurrence threshold of 0.84 (calculated from the discovery cohort of about 550).
67	Inter_WSI_g2g2_patch_cooccur_low	Cooccurrence of grade 2 and grade2 patches (size 123 micro m) below a threshold in windows (size 369 micro m). cooccurrence threshold of 0.84 (calculated from the discovery cohort of about 550).
68	Inter_WSI_g2g3_patch_cooccur_high	Cooccurrence of grade 2 and grade 3 patches (size 123 micro m) above a threshold in windows (size 369 micro m). cooccurrence threshold of 1.49 (calculated from the discovery cohort of about 550).
69	Inter_WSI_g2g3_patch_cooccur_low	Cooccurrence of grade 2 and grade 3 patches (size 123 micro m) below a threshold in windows (size 369 micro m). cooccurrence threshold of 1.49 (calculated from the discovery cohort of about 550).
70	Inter_WSI_g3g3_patch_cooccur_high	Cooccurrence of grade3 and grade3 patches (size 123 micro m) above a threshold in windows (size 369 micro m). cooccurrence threshold of 2.54 (calculated from the discovery cohort of about 550).
71	Inter_WSI_g3g3_patch_cooccur_low	Cooccurrence of grade 3 and grade 3 patches (size 123 micro m) below a threshold in windows (size 369 micro m). cooccurrence threshold of 2.54 (calculated from the discovery cohort of about 550).
72	Ratio_inter_WSI_g1g1_patch_cooccur_low_high	Inter_WSI_g1g1_patch_cooccur_low/ Inter_WSI_g1g1_patch_cooccur_high

G: grade, p:pleomorphism. TILs: tumour infiltrating lymphocytes.

Supplementary Table (3) Mean and standard deviation (Mean ± SD) of heterogeneity score in the different pathological parameters

Pathological parameters	Mean ± SD of heterogeneity score
**Tumour size (cm)**	
≤2	4.5 ± 17
>2	19.8 ± 31.1
**Tumour Grade**	
Grade 1	2.9 ± 17.9
Grade 2	7.4 ± 22.3
Grade 3	15. 4±15.7
**Histologic tumour types**	
No Special Type (NST)	12.5 ± 25.2
Lobular	4.4 ± 25.1
Other special types	0.39 ±15.8
Mixed subtypes	11.1 ± 25.9
**Lymph node status**	
Negative	0.39 ± 15.8
Positive	11.1 ± 25.9)
**Lymhovascular invasion**	
Absent	5.3 ± 27.3
Present	7.2 ± 21.5
**ER expression levels.**	
Lower <10%	9.4 ± 21.6
Intermediate (10-99%)	8.3± 21.5
Highest (100%)	7.1 ± 22.1
**PR status**	
Low ≤10%	11.5 ± 24.7
High>10%	6.9 ± 24.1
**Ki67 expression**	
Low ≤10%	2.0 ± 17.7
High >10%	11.9 ± 23.3

ER: estrogen receptor, PR Progesterone receptor

Supplementary Table (4) Multivariate analysis of overall intra-tumour heterogeneity score in luminal breast cancer with prognostic parameters

Parameters	BCSS			DMFS		
	Hazard ratio	95% CI	*P-value*	Hazard ratio	95% CI	*P-value*
Heterogeneity score	2.2	1.3-3.5	***0.001***	2.3	1.5-3.5	***<0.0001***
Tumour size	1.3	0.8-1.9	*0.31*	1.3	0.8-2.1	*0.17*
Grade	2.6	1.8-3.9	***<0.0001***	1.8	1.3-2.5	***0.001***
LVI	1.6	0.9-2.6	*0.062*	1.5	1.5-3.7	***<0.0001***

BCSS, breast cancer specific cancer, DMFS, distant metastasis free survival, LVI, lymphovascular invasion. 95%CI, 95% confidence interval. Significant p values are in **bold**.

SUPPLEMENTARY FIGURES

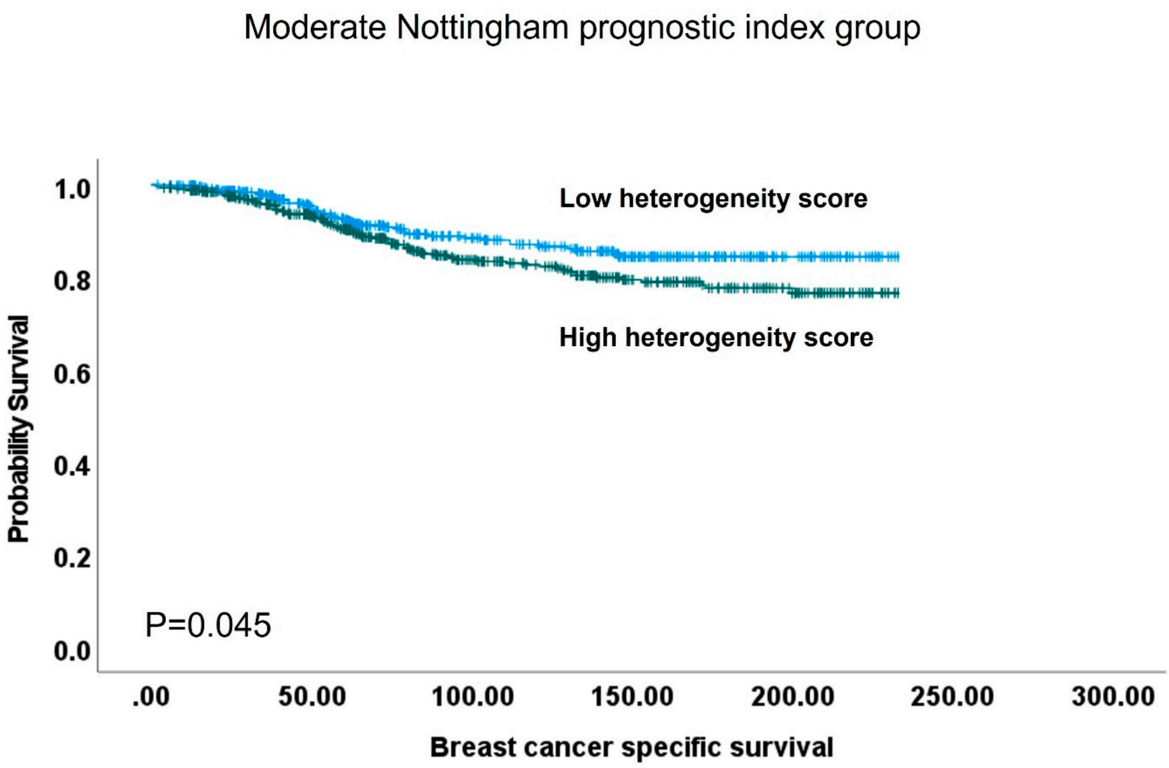

Supplementary figure 1: shows Kaplan Meier association of heterogeneity score with breast cancer-specific in intermediate risk patients (moderate Nottingham prognostic index).

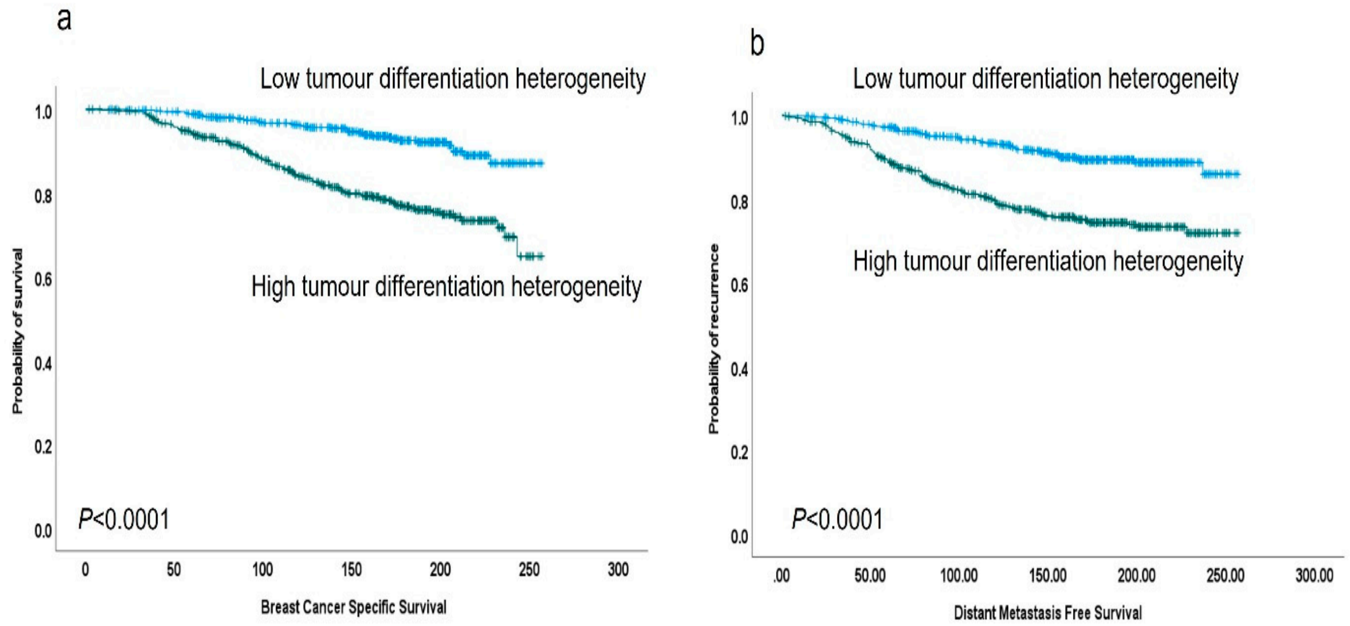

Supplementary figure 2: shows Kaplan Meier association of tumour differentiation heterogeneity (high vs low) with breast cancer-specific survival.

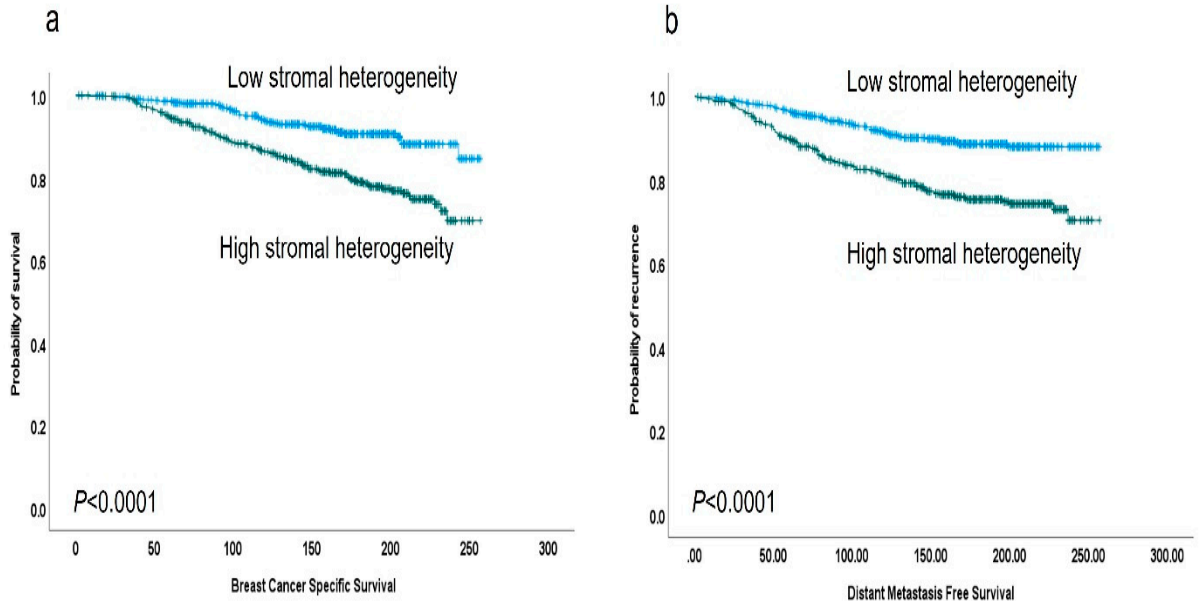

Supplementary figure 3: shows Kaplan Meier association of stromal heterogeneity (high vs low) with breast cancer-specific survival.

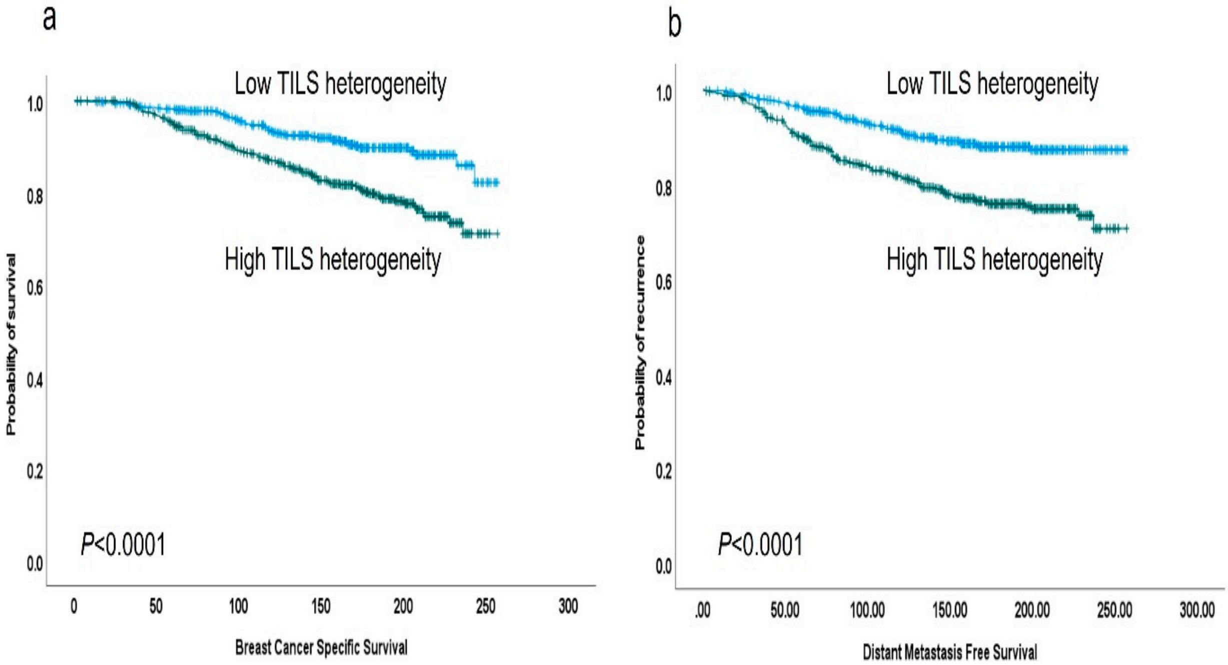

Supplementary figure 4: shows Kaplan Meier association of tumour infiltrating lymphocytes (TILS) heterogeneity (high vs low) with breast cancer-specific survival.
